# Composite amine mixed matrix membranes for high-pressure CO_2_-CH_4_ separation: synthesis, characterization and performance evaluation

**DOI:** 10.1098/rsos.200795

**Published:** 2020-09-09

**Authors:** Nur Aqilah Bt Fauzan, Hilmi Mukhtar, Rizwan Nasir, Dzeti Farhah Bt Mohshim, Naviinthiran Arasu, Zakaria Man, Hafiz Abdul Mannan

**Affiliations:** 1 Department of Chemical Engineering, Universiti Teknologi PETRONAS, Seri Iskandar, 32610 Perak, Malaysia; 2 Department of Petroleum Engineering, Universiti Teknologi PETRONAS, Seri Iskandar, 32610 Perak, Malaysia; 3 Department of Chemical Engineering, University of Jeddah, Jeddah 23890, Saudi Arabia; 4 Institute of Energy and Environmental Engineering, University of the Punjab, 54590 Lahore, Pakistan

**Keywords:** high-pressure separation, composite mixed matrix membranes, CO_2_-CH_4_ separation, Maxwell model, performance prediction

## Abstract

The key challenge in the synthesis of composite mixed matrix membrane (MMMs) is the incompatible membrane fabrication using porous support in the dry–wet phase inversion technique. The key objective of this research is to synthesize thin composite ternary (amine) mixed matrix membranes on microporous support by incorporating 10 wt% of carbon molecular sieve (CMS) and 5–15 wt% of diethanolamine (DEA) in polyethersulfone (PES) dope solution for the separation of carbon dioxide (CO_2_) from methane (CH_4_) at high-pressure applications. The developed membranes were evaluated for their morphological structure, thermal and mechanical stabilities, functional groups, as well as for CO_2_-CH_4_ separation performance at high pressure (10–30 bar). The results showed that the developed membranes have asymmetric structure, and they are mechanically strong at 30 bar. This new class of PES/CMS/DEA composite MMMs exhibited improved gas permeance compared to pure PES composite polymeric membrane. CO_2_-CH_4_ perm-selectivity enhanced from 8.15 to 16.04 at 15 wt% of DEA at 30 bar pressure. The performance of amine composite MMMs is theoretically predicted using a modified Maxwell model. The predictions were in good agreement with experimental data after applying the optimized values with AARE % = ∼less than 2% and *R*^2^ = 0.99.

## 1. Introduction

Generally, the high-grade gas separation membranes for industrial applications should possess superior separation performance with outstanding permeability, which has the potential to overcome the trade-off relationship of the Robeson upper bound for polymeric membrane [1]. Moreover, membranes that are mechanically stable, cost-effective in production, and able to withstand high pressure and temperature are desirable [2,3]. To achieve these criteria, the membrane materials and structures are the critical decision as they have a profound influence on the entire membrane performance [4]. Thus, it is very important to develop a new combination of membrane materials that meet industrial requirements.

Attempts are being made by researchers to incorporate inorganic fillers such as zeolite, metal-organic framework (MOF), carbon molecular sieve (CMS), and other fillers into a polymer matrix to develop mixed matrix membranes (MMMs). The MMMs are reported to have higher gas permeabilities with recuperated gas selectivity contrary to respective pure polymer membranes [5,6]. Recently, Cheng *et al.* showed a substantially improved CO_2_-CH_4_ selectivity and permeability by adding coated size-selective MOF cores with a covalent organic framework (COF) in polysulfone (PSF) with a thickness of 40–70 µm [7]. They reported 48% and 79% enhancements in CO_2_ permeability and CO_2_-CH_4_ selectivity, respectively, in comparison with that of neat PSF from 11.3 Barrer to 22.9 Barrer. Nonetheless, the CO_2_ permeability of 22.9 Barrer is still below the standard for cost-effective CO_2_ capture and Robeson upper bound curve. When the thickness of MMM is above 10 µm [8,9], this will consequently cause low CO_2_ permeability of 0.1–22.9 Barrer and high cost of membrane fabrication. Thus, MMMs need to be made from a thinner selective layer to achieve the industrial demand for elevated product yield, which means high gas flux.

Nevertheless, the majority of MMMs are dense membranes with thickness exceeding 10 µm as a result of complication in preparing fillers of this proportion and in averting particle agglomeration during membrane fabrication [10–12]. The thin-film composite membrane (TFCM) is being introduced to overcome the challenges mentioned above. TFCMs comprise a minimum of two layers, i.e. the support layer and the selective layer [13,14]. Additionally, there may be other layers such as the gutter layer to diminish pore penetration and protective layer to overcome pore defects [15]. Norahim *et al*. developed TFCM by incorporating graphene oxide (GO) in polyethylene glycol (PEG 400)/polyether block amide (Pebax 1657) blended polymer to form a selective layer on polyetherimide (PEI) with a non-woven backing support layer (Novatexx 2470) [16]. The 50 : 50 ratio of PEG/Pebax membrane, including the non-woven support layer, resulted in a double increment of permeance and CO_2_-CH_4_ selectivity compared to pure Pebax 1657. In another work, Khalilinejad *et al*. also prepared three-layer TFCM of Pebax 1657, polyvinyl chloride (PVC) with non-woven support layer, and reported that the CO_2_ permeance and CO_2_-CH_4_ selectivity of the membrane increased upon pressure increment with 16.7% and 21% in contrast to the pure Pebax 1657 [17]. Furthermore, Mozafari *et al*. reported the use of a non-woven support layer with polymethylpentyne (PMP) and 2 wt% UiO-66-NH_2_ and formed a defect-free boundary which managed to increase CO_2_-CH_4_ selectivity from 15.9 to 35.3 at 5 bar [18].

Several modelling approaches have been developed to minimize the time-consuming and costly experiments and to predict the performance of MMMs and MMMs with a third component [19–23]. On the other hand, the performance of membranes depends upon the operating parameters like pressure and temperature. As the pressure difference between feed and permeate side is large, the membranes exhibit high permeability [24]. Therefore, it is necessary to model the effect of pressure on the performance of MMMs. In the literature, very limited studies are reported to address the pressure dependency of gas transport. Maghami *et al*. [21] modified the Van't Hoff-Arrhenius equation to model the transport of gas through MMM, using the corresponding experimental data at 35°C in the feed pressure range from 2 to 12 atm. They found 5.1% AARE for 300 data points.

In our previous work, we have reported high CO_2_ permeance and CO_2_-CH_4_ selectivity up to 117.32 GPU and 20.21, respectively, polyethersulfone (PES) with different concentrations of DEA and fixed loading of carbon molecular sieve (CMS) [25]. However, these membranes were not tested at high pressure due to the possibility of fracture of the membrane in high pressure without any porous support layer. Building on this earlier work, in this study, we have added a non-woven support layer at the bottom layer of the amine MMM. To the author's best knowledge, no study on the synthesis and performance analysis of composite amine MMM at high pressure is available. Moreover, there is no study reported in the literature on the performance prediction of composite amine mixed matrix membranes. Therefore, the amine composite mixed matrix membrane is developed and characterized for high-pressure application up to 30 bar. The permeance and ideal selectivity of the synthesized MMMs were investigated. Also, the Maxwell Model was used because it provides the exact solution for the permeability of randomly distributed and non-interactive homogeneous solid spheres in a continuous matrix [26,27]. The model is modified by considering the effect of high pressure (10–30 bar), filler loading (10 wt%), and amine concentration (15 wt%) on the carbon dioxide transport properties of PES-CMS-DEA composite amine MMMs.

## 2. Experimental procedures

### 2.1. Materials

The flaked form of PES polymer (ULTRASONE E-6020P) was purchased from BASF Germany. The average molecular weight of PES was 50 000 g/mol. PES was the main polymer for polymeric membranes fabrication in this study because of its advantageous properties, including chemical, thermal and mechanical stability [28]. The glass-transition temperature (*T_g_*) for PES was 225°C. The solvent *N*-methyl-2-pyrrolidone (NMP) used for this work was purchased from Merck, Germany, with informed purity of 99.99%. NMP was used as a solvent because of its high chemical stability, solubility, low flammability, boiling point, and low volatility, which is good for the homogeneous membrane's outcome. The CMS is used as an inorganic filler in the composite MMMs synthesis, and it allowed fast transport of gas molecules and had a uniform pore size on the surface [29]. It was acquired from Enviro Chemicals, Japan. DEA with 99% purity was purchased from Merck. DEA is the most extensively used secondary amine for the removal of acid gases, due to its favourable reaction kinetics and resistance to solvent degradation [30]. The support layer used at the bottom of the composite amine mixed matrix membrane was a Novatexx 2471, acquired from Freudenberg Filtration Technologies, Germany.

### 2.2. Methods

Five different types of membranes were synthesized in this study. The list of membranes synthesized, along with their concentrations, is tabulated in table 1. The polymer concentrations reported in this study were selected based on dope solution viscosity which has been studied in our previous study [31].

**Table 1.** Membrane samples composition.

polymer concentration (wt%) polyethersulfone (PES)	CMS loading (wt%)	DEA amine concentration (wt%)	membrane ID
20	0	0	CM
20	10	0	CM-C10
20	10	5	CM-C10D5
20	10	10	CM-C10D10
20	10	15	CM-C10D15

CM, composite membrane; C, carbon molecular sieve; and D, di-ethanolamine.

#### 2.2.1. Synthesis polyethersulfone composite membrane

The procedure for the synthesis of pure PES composite membrane consists of (i) dispersion of the PES in NMP, (ii) casting of the solution onto a non-woven fabric, and (iii) successive dry–wet phase inversion in the non-solvent water bath. The based PES composite membrane was synthesized by using 20 wt% dried PES of solvent basis at critical polymer concentration. The dried PES was added into the NMP solvent and stirred (100 rpm) for 24 h at room temperature. The entrapped air bubbles in the solution were removed by degassing in the Sonication bath (Transsonic Digital S, Elma) for 1 h. Membranes were cast using polypropylene, non-woven fabric as a support layer on a flat, dust-free, dry, smooth, glass plate. Water and acetone were used to wash the glass plate and remove the impurities. Dust particles were removed from the glass plate by using compressed air. The mixed polymer solution was transferred onto the glass plate with an adjusted casting knife thickness of 200 µm. Then, the casting knife was relocated over the solution to form the membrane's uniform layer by using a film applicator. The prepared membrane film was dried for about 60 s at room temperature by dry phase inversion before it was immersed in water for wet phase inversion. The wet phase inversion technique used water as a non-solvent [32] to convince a chain of liquid–liquid phase separations [33]. As the coagulation step completed, the TFCM was washed using distilled water and placed inside the room temperature condition to left dry. The synthesized membranes were to be used for characterization.

#### 2.2.2. Synthesis of composite mixed matrix membrane

The procedures of casting composite mixed matrix membrane consisted of (i) dispersion of the CMS particles in NMP solvent, (ii) sonication of the solution to keep particles in suspension, (iii) mixing of PES with CMS solution, (iv) casting of the solution onto a non-woven fabric and (v) successive dry–wet phase inversion in the non-solvent water bath. The 10 wt% CMS particles were added in NMP and stirred for 15 min. The CMS-NMP solution was ultrasonicated for 1 h at a frequency of 100 Hz. 20 wt% of dried PES on a solvent basis was added to the solution and stirred for 24 h at room temperature. The entrapped air bubbles in the solution were degassed for 1 h. After degassing, the mixed matrix membrane solution was cast as described in §2.2.1. The prepared membrane film was dried for about 60 s by dry phase inversion before it was immersed in water for wet phase inversion. As the coagulation step completed, the TFCM was washed using distilled water and placed inside the room temperature condition to dry.

#### 2.2.3. Synthesizing of composite amine mixed matrix membrane

**Figure 1.**
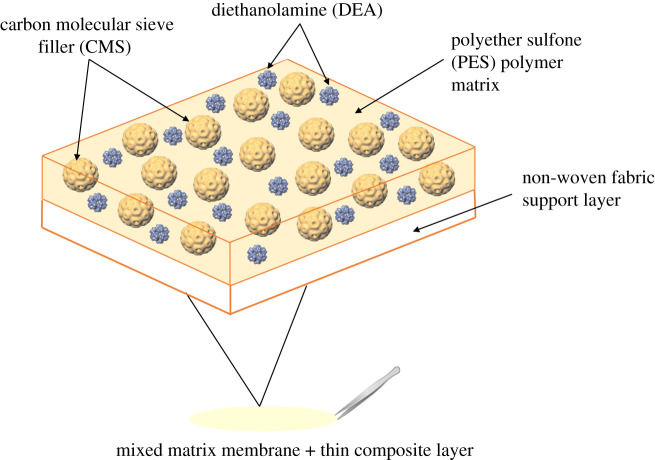
Representation of amine composite mixed matrix membranes.

**Figure 2.**
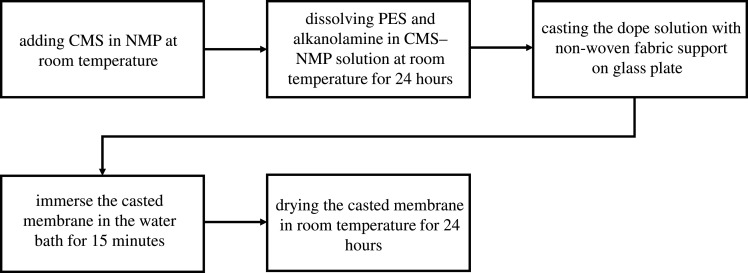
Process for the membrane's preparation methodology.

Three composite amine mixed matrix membranes were synthesized by using the same methodology, as discussed in §2.2.2. The procedures of casting composite amine mixed matrix membrane consist of (i) dispersion of the CMS particles in NMP solvent, (ii) sonication of the solution to keep particles in suspension, (iii) mixing of PES and DEA with CMS solution, (iv) casting of the solution onto a non-woven fabric and (v) successive dry-wet phase inversion in the non-solvent water bath. The 5 wt%, 10 wt% and 15 wt% amine compositions based on the polymer weight were used in this study. The schematic illustration of amine composite mixed matrix membranes is shown in figure 1. The process for membrane development is visualized in figure 2.

### 2.3. Characterization of membranes

The synthesized membrane had been analysed for physico-chemical characterization. Listed below are the physico-chemical properties of membranes to be characterized and analysed. The variable pressure field emission scanning electron microscopy (VPFESEM) is used to study the morphology of the composite membrane with polypropylene non-woven fabric evaluated. The Fourier transform infrared (FTIR) is used to examine the functional group in the composite membrane between polymer components and inorganic materials. The differential scanning calorimetry (DSC) is used to find the glass transition temperature (*T_g_*) of membranes. It provides a qualitative analysis of the flexibility or rigidity of the polymer chain. The effect of polypropylene non-woven fabric on mechanical properties of amine mixed matrix membrane was evaluated by universal testing machine (UTM).

### 2.4. Gas separation analysis

**Figure 3.**
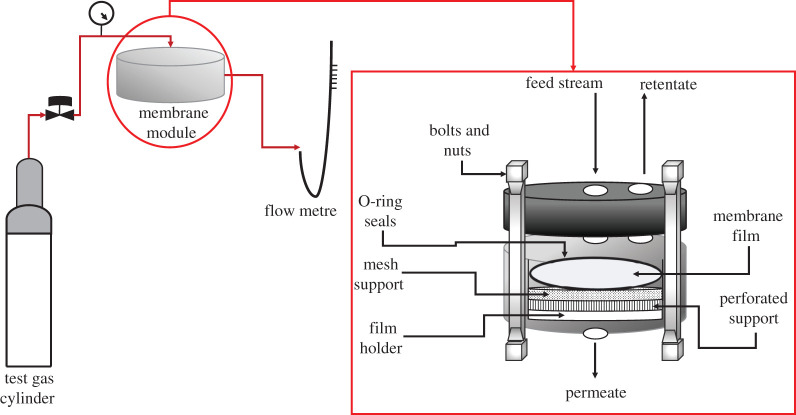
Gas permeation test set up.

The membranes were tested for the permeance of CO_2_ and CH_4_ (99.99% purity), using a gas permeation unit at a feed pressure of 10, 15, 20, 25 and 30 bar at room temperature (25°C). The gas permeation unit is described as a 17.35 cm^2^ two-compartment cell. The membrane was arranged on a porous support, holding O-rings between two flanges. The unit was vacuumed to eliminate any residual gases. Permeate pressure was constant at atmospheric pressure, P atm. The volumetric flow rate of permeate gas streams was calibrated using a bubble flow metre. Figure 3 represents the schematic set up of the gas permeation equipment [25,34].

A bubble flow metre which can detect flow rates as low as 100 ml mi^−1^ was used to measure the permeate rate of gas streams. The equation used to calculate the gas permeance in GPU (1 GPU = 1 × 10^−6^ cm^3^ (STP)/s.cm^2^.cmHg) is as follows:

2.1 $$\frac{P_{i}}{L}=\frac{J_{i}}{\Delta p_{i}}$$

where *L* is the membrane thickness (cm), $J_{i}$ represents the gas flux (cm^3^ cm^−2^ s), whereas $\Delta p_{i}$ is the transmembrane pressure (cmHg). Instead, the selectivity of the membrane was calculated by the following equation:

2.2 $$\alpha_{CO_{2}/CH_{4}}=\frac{P_{CO_{2}}}{P_{CH_{4}}}.$$

## 3. Results and discussion

### 3.1. Morphological analysis of synthesized composite mixed matrix membranes

The morphological analysis is carried out for all synthesized membranes, and for the ease of understanding discussion about morphologies of pure PES, composite mixed matrix membranes and amine composite mixed matrix membranes are divided into subsections. As described in the above section, the synthesis of all composite membranes has been carried out by phase immersion precipitation by using water as a non-solvent. It is, therefore, necessary to understand the effect of the fabrication technique on the membrane structure because the membrane structures are a major contributor to the efficiency of membrane separation [35,36]. The membrane structure may be categorized based on polymer, solvent, and non-solvent combination. In the cited literature, it is mentioning that the membranes developed by a combination of solvent (NMP)/(non-solvent water) have a finger-like and spongy structure [37,38]. All developed composite membranes exhibit the finger-like and spongy structure. Therefore, for ease of understanding, the explanation of the structures has been discussed here.

#### 3.1.1. Morphological analysis of pure polyether-sulfone composite membranes

**Figure 4.**
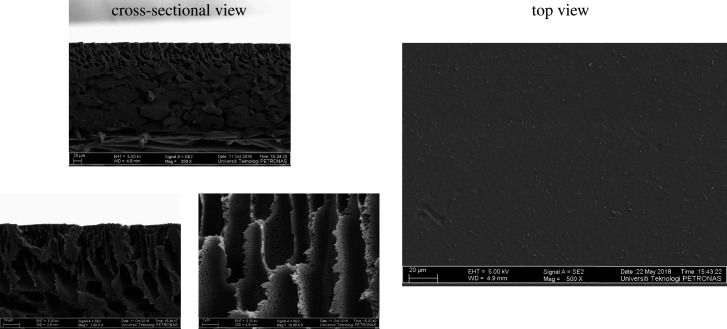
Cross-sectional and top view of pure PES composite membrane.

The top and the cross-sectional view of the PES composite membrane was analysed by FESEM, as visualized in figure 4. It is observed that the surface of the developed membrane is smooth, dense and defect-free. The cross-sectional view of the PES composite membrane has shown the asymmetric structure, which was composed of non-woven fabric with a finger-like and spongy structure. The developed pores in finger-like structure are sponge pores. Moreover, the top view of the pure PES membranes is smooth and non-porous. The structure was produced because of the immediate phase inversion between solvent and non-solvent in the coagulation bath. Besides, the strong affinity between NMP and water caused the formation of such a structure [39,40], and this type of structure affects the mechanical strength compared to a membrane structure containing macro voids [41].

#### 3.1.2. Morphological analysis of composite mixed matrix membranes

**Figure 5.**
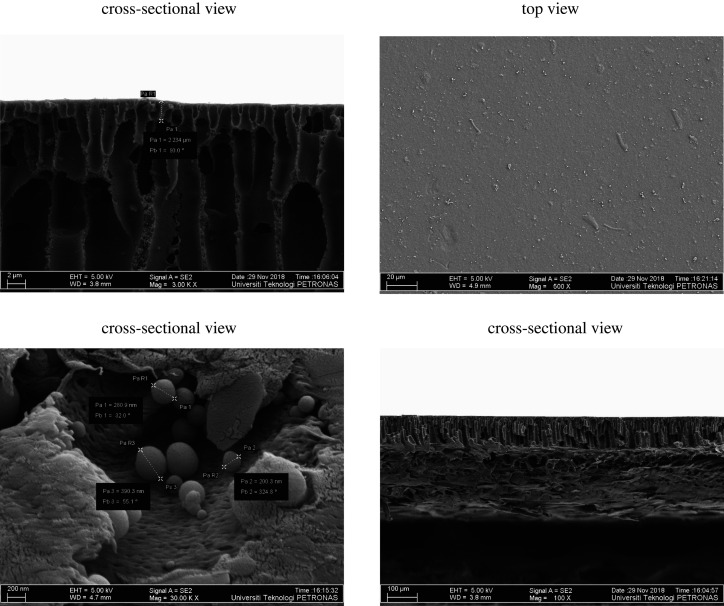
Top and cross-sectional view of composite mixed matrix membrane CM-C10.

The top and cross-sectional micrographs of CMS (10 wt%) loaded polyethersulfone composite membrane (CM-C10) are shown in figure 5. The top view of the developed membrane is smooth and without any pinholes and micro-voids. The cross-sections of supported CM-C10 composite membranes exhibit the 183.1 µm thickness of non-woven fabric support and 93.86 µm thickness of CM-C10 above the support. Good compatibility between CM-C10 and non-woven fabric support was also observed. Moreover, in the cross-section micrographs, the CMS particles are well distributed with very little agglomeration.

The smooth surface of CM-C10 is due to the carbon-based structure of CMS, the dispersion of CMS is good enough, and no agglomeration was observed on the surface of the CM-C10 mixed matrix membrane. Moreover, the absence of cracks on the surface shows that the developed CM-C10 is not brittle and has good stability [42]. The cross-sections of CM-C10 exhibit the typical asymmetric porous structure with no microvoids. The dispersion of the CMS caused competition between the shearing forces that were applied to break the agglomeration and the coherent force that keeps the agglomerate from dispersing. Therefore, the cohesive force that arises from the Vander Walls interparticle interaction between CMS molecular sieves becomes dominant at higher loading than the shearing forces that attempted dispersion of CMS particles [43]. These results are supported by previous studies where the structure of fabricated membrane samples showed a high similarity [44,45].

#### 3.1.3. Morphological analysis of composite amine mixed matrix membranes

**Figure 6.**
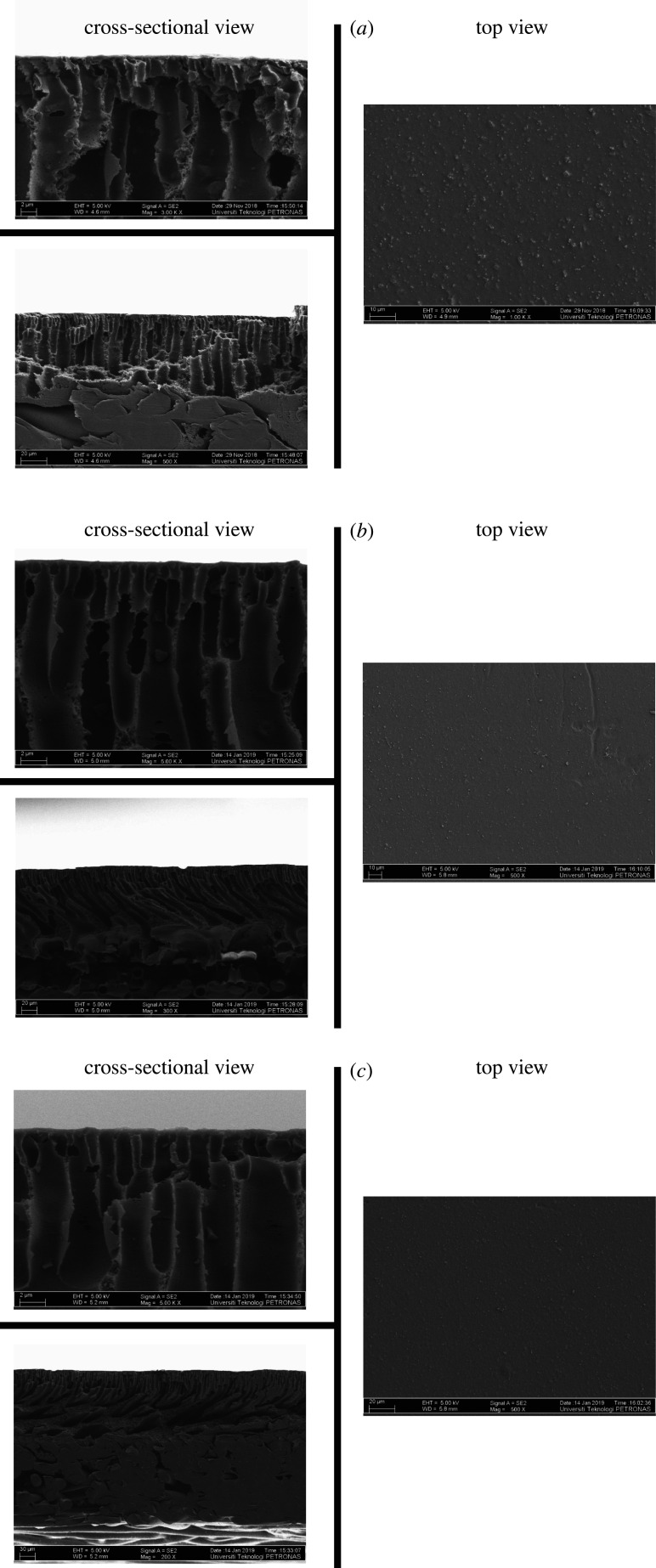
Top and cross-sectional view of amine composite MMMs with 10 wt% CMS and (*a*) 5 wt% DEA, (*b*) 10 wt% DEA and (*c*) 15 wt% DEA.

The top and cross-sectional morphology of ternary composite amine mixed matrix membranes with 5 wt%, 10 wt% and 15 wt% DEA and 10 wt% CMS is given in figure 6*a*–*c*. All top views are smooth and defect-free and cross-sectional micrographs exhibit the typical finger-like and sponge-like pore structures, which help to reduce the transport resistance and to provide enough mechanical strength, respectively. The cross-sections of CM-C10D5, CM-C10D10 and CM-C10D15 exhibit the 203.0 µm thickness of non-woven fabric support and 34.99 µm, 126.90 µm and 137.50 µm thickness of CM-C10D5, CM-C10D10 and CM-C10D15, respectively, above the support. It was also observed at higher magnifications that by the addition of low molecular DEA, the small agglomeration which appeared in CM-C10 membranes had disappeared, and CMS is well dispersed in the PES matrix. The good dispersion of CMS by the addition of DEA is because of the good interaction and solubility of PES, NMP and DEA. DEA helped CMS particles to be wetted and surrounded better with PES chains. It is observed that with the addition of DEA, the CMS particles have not shown apparent defects or agglomeration and void formation. It was feasible to make interfacial void-free CMS-filled glassy polymer membranes by filling the CMS-polymer interface with a low molecular weight third component, which could interact simultaneously with polymer and CMS. The strongest interaction can be hydrogen bonding between polymer and the third component [46]. The absence of phase separation in the PES-CMS-DEA membrane is due to the solvency of the third component in the solvent during the dope solution preparation, as suggested by Yong *et al*. [46]. As the DEA also belongs to a low molecular weight additive family, the addition of DEA makes the dope solution less stable (thermodynamically), which causes rapid demixing in the coagulation bath. The nature of the additive may affect the exchange rate of solvent and non-solvent during phase inversion process and influence the kinetic of precipitation and formation of resulting membrane morphology [47]. The change in kinetics has a negligible effect because a very low amount of additive has been added to cause a change in the viscosity of the dope solution [48]. Thus, it is expected that the presence of DEA, improved membrane structure and non-woven support will enhance the performance of the synthesized membranes at high pressure.

### 3.2. Spectral analysis

The analysis is carried out to assess the possible change in polymer (PES) structure or any chemical interaction due to the addition of DEA and inorganic filler CMS. The FTIR analysis of all developed composite membranes is recorded and shown in electronic supplementary material, figure S1. It was observed that all spectra are almost identical to each other and concluded that there is no chemical interaction between PES and DEA. It is also observed that by the addition of CMS, there is no significant change in the PES spectra, which confirms the physical attachment of the polymer chain to CMS. But by the addition of DEA, there were small increases or decreases in the band frequencies, as shown in electronic supplementary material, figure S1. The explanations of the functional group of the possible peaks are tabulated in table 2. The findings of this study are similar to the previously cited literature [25,50,51].

**Table 2.** Possible FTIR spectra bonds in developed composite membranes [49].

possible spectra bonds	band frequency (cm^−1^)
C=C aromatic	1575.18
C=O ketone	1730.08
benzene/ aromatic rings	1484.00
sulphone oxide	1020.55
C-O-C ether	1139.65
−CH_2_ bending	2928.55
−O-H	3349.99
−N-CH_3_	2928.55

### 3.3. Thermal analysis

#### 3.3.1. Differential scanning calorimetry analysis

DSC analysis was carried out to study the effect of CMS and DEA addition on the glass transition temperature of synthesized membranes. The glass transition temperature of neat CM, CM-C10 and CM-C10D (5–15) with fixed loading of CMS (10 wt%) and various concentrations of DEA (5–15 wt%) composite membranes was tabulated in table 2. It was observed that at lower loading of CMS (10 wt%), the *T_g_* is decreased. This may be due to an increase in free volume and chain mobility around CMS. Thus, the *T_g_* of the PES shifts to lower temperatures with the addition of CMS particles [52,53].

**Figure 7.**
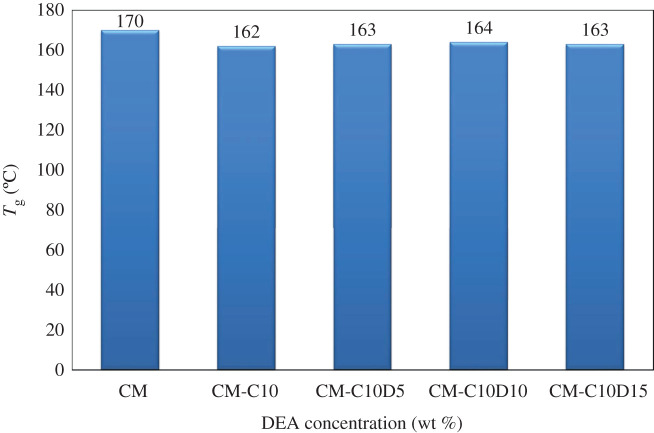
Glass transition temperatures of developed composite membranes.

Figure 7 shows the effect of DEA on *T_g_.* It was noted that the decrease of about 6–8°C below that of pure PES composite membrane (CM) at various concentrations of DEA (5–15 wt%) was observed. The *T_g_* of CM-C10D (5–15 wt%) amine composite MMM is lower than the *T_g_* of pure PES composite membranes. It is also observed by the addition of DEA in membranes, and the *T_g_* is reduced. This shows that membranes are less crystalline and have an amorphous structure. The addition of DEA decreases the value of *T_g_* and increases the permeance of the membranes. It is believed that DEA is a low molecular weight organic solvent added to a polymer matrix to modify its physical properties such as flexibility (by lowering the glass transition temperature) and microstructure. The improvement of the structure has been confirmed through morphological analysis. Furthermore, DEA is relatively small as compared to that of the polymer molecule, and henceforth can easily penetrate the polymer matrix and enhance the interaction of membrane phases, and may reduce the cohesive forces operating between the polymer chains increasing the chain segmental mobility [54]. The variation of *T_g_* has been correlated with the gas permeability for different concentrations of additive, which will be discussed in §3.5.2. Therefore, it can be concluded that the addition of a DEA to the formulation of a mixed matrix membrane strongly affects its final structure. Sen *et al*. also found a similar trend for polycarbonate-zeolite 4A and pNA membranes. They found that the addition of pNA in PC-zeolite membranes resulted in a reduction in *T_g_* values compared to pure PES membranes. They concluded that pNA provided the interaction between polymer and zeolite. Thus, according to the above study, DEA acted as an arbitrating agent to provide the interaction and is essential for CMS to affect the PES polymer matrix [55]. For all types of membranes, whether PES-CMS or PES-CMS-DEA membranes, a single *T_g_* was observed, which points to the presence of a single homogeneous phase in membranes.

### 3.4. Mechanical analysis

The mechanical properties of selected membranes were determined by tensile testing from the universal testing machine (UTM). Young's modulus and tensile strength are summarized in table 3. It was observed that by the addition of CMS, Young's modulus values are decreased from 1280 MPa to 412 MPa, which is most likely due to the stress concentration tempted by CMS aggregation at 10 wt% CMS loading. Similar findings have been observed by Manawi *et al*. [56]. On the other hand, with the addition of 5 wt% a much lower reduction (approx. 11.36%) in Young's modulus was observed, which may be due to any agglomeration of CMS. It was also observed in table 3 that the highest elastic stiffness Young's modulus value is 3278 MPa with the addition of CMS and DEA in the polymer matrix.

**Table 3.** Mechanical properties of synthesized composite membranes.

membrane	Young's modulus (MPa)	tensile strength (MPa)
CM	1280	11.94
CM-C10	412	12.92
CM-C10D5	1136	20.08
CM-C10D10	3278	17.25
CM-C10D15	—	16.72

Moreover, the results appeared to be independent of the loading of CMS and concentration DEA. However, Young's modulus values are independent but with the addition of 10 wt% DEA Young's modulus value is increased from 1280 MPa to 3278 MPa. The increase in Young's modulus is also due to the increase of polymer chain entanglement with CMS and DEA contents, which leads to constrained polymer chain mobility resulting in a stiffer film. It is further strengthened by the presence of polar -SO_2_ −, DEA group and rigid aromatic ring in PES backbone. It seems to suggest that Young's modulus is likely to be underestimated from the tensile test. This claim was highlighted in previous literature [57,58].

The tensile strength is increased from 11.94 MPa to 12.92 MPa, which suggested that the addition of CMS increased the membrane strength. The increase of membrane strength may be due to restriction of chain segmental mobility by the addition of CMS [59]. It was also observed that with the addition of different concentrations of DEA, the tensile strength is enhanced as compared to pure PES membranes. This indicates that DEA is well mixed within the dope solution and effectively improves the mechanical properties of PES-CMS-DEA composite mixed matrix membranes. The improved mechanical properties confirm the strong interaction between the components of dope solution [60]. Moreover, the decrease in tensile strength with the increase of DEA concentration may be due to an increase in the flexibility of the polymer chain. The decrease in tensile strength by the increase of DEA concentration is due to the increased porosity of the developed PES/CMS/DEA composite mixed matrix membranes [47,61]. Similar findings have been observed by Ali *et al*. [62] in cellulose acetate/glycol/zinc oxide blend membranes. However, the PES/CMS/DEA composite membranes have higher tensile strength than pure PES and PES/CMS composite mixed matrix membranes. These improvements in mechanical properties of the membrane may be attributed to the strong interactions between the polyethersulfone matrix, CMS, and DEA and homogeneous dispersion and adhesion of CMS as observed by microscopy measurements.

### 3.5. Gas performance analysis

#### 3.5.1. Effect of pressure on the gas separation performance

**Figure 8.**
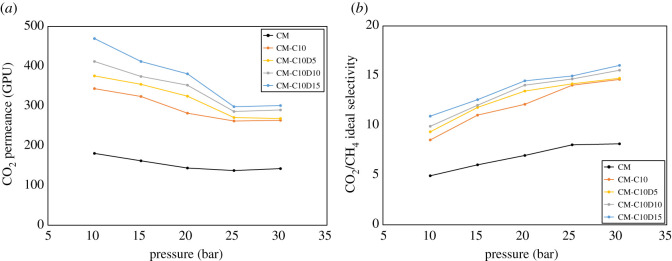
(*a*,*b*) Effect of pressure on the gas separation performance of amine composite mixed matrix membranes.

The effect of pressure (10–30 bar) on the carbon dioxide permeance and CO_2_-CH_4_ selectivity through composite and ternary composite mixed matrix membranes has been shown in figure 8*a*,*b*. The presented values have been calculated by equations (2.1) and (2.2). The figures exhibit a reduction in permeance and an increase in selectivity with the increase in pressure. In figure 8*a*, the maximum permeance is at a lower pressure of 10 bar, and as pressures approach 30 bar, the permeability is moved towards minimum value. The percentage decrease was in the range of 17.89% ± 1 to 35.90% ± 1 for all developed pressures across the entire pressure range. Figure 8*b* shows the opposite trend of CO_2_-CH_4_ selectivity. The maximum selectivity value (16.04) was at 30 bar pressure. In the cited literature, the plasticization pressure of PES is approximately 28 bar [63]. Therefore, to investigate the effect of DEA on the plasticization, the 30 bar pressure was selected. On the other hand, for all membranes, a decrease in CO_2_ permeability with elevated pressure is observed up to 25 bar, which indicates that there is no significant plasticization [64]. After the gas had passed the plasticization pressure, it was observed that the permeance was slightly increased. This increase is due to slight plasticization [65,66]. Thus, it is concluded that the DEA has a minor effect on plasticization after the plasticization pressure of the PES. The decrease in permeance before the plasticization pressure is due to competitive sorption, sorption isotherm concave shape [67,68]. It constitutes a reduction in the driving force of transport with increasing pressure and a gradual saturation of the material which may cause lower mobility [69].

#### 3.5.2. Synergetic effect of carbon molecular sieve and diethanolamine addition on gas performance

**Figure 9.**
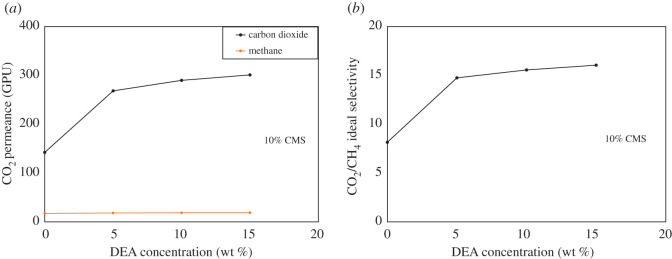
(*a*,*b*) Effect of DEA concentration on gas separation through amine composite MMMs.

The synergetic effect of carbon molecular sieve and DEA on the permeability and ideal selectivity of composite amine mixed matrix membranes at a pressure of 30 bar has been shown in figure 9*a*,*b*. The focus of this study was to investigate the effect of different concentrations of DEA with a fixed loading of CMS. According to the hypothesis, it was observed in the developed composite ternary MMMs that CO_2_ permeance (142.21–300.84 GPU) and CO_2_-CH_4_ selectivity (8.15–16.04) increased by increasing the concentration of DEA as shown in figure 9*a*,*b*. By contrast, there was a very small change in the value of CH_4_ permeance with the addition of DEA and CMS.

It was also observed that the loading of CMS was also one contributor to performance enhancement. In figure 9*a*, it was also observed that carbon dioxide permeance is higher than methane permeance. The pore size of CMS might be the reason for this occurrence. The kinetic diameter of CO_2_ (3.3 Å) is smaller than the pore size of the CMS, whereas the kinetic diameter of CH_4_ (3.8 Å) is bigger than the pore size of CMS. Therefore, it could hinder CH_4_ molecules from passing through the membrane [70]. CMS gas transport is less resistive than the PES matrix and offers the most selective route since CMS can distinguish between the size and shape of gas penetrators. Conversely, the gaps or voids enable the bypass of gas through its non-selective and non-resistive pathways, and it is presumed to be the Knudsen diffusion [71].

The increase in the permeance and selectivity is due to ‘like dissolves like phenomena', as the DEA is acting as a facilitator for the CO_2_ transport through composite ternary MMMs. The DEA also has a good affinity with carbon dioxide and a negligible affinity with methane gas. The escalation in CO_2_ permeance with increasing DEA concentration was simply due to the availability of more amine for CO_2_ transport. Upon CO_2_-amine reaction, various ionic species are formed, such as carbamates, protonated amines and zwitterions. Another possible reason is that these ion species have occupied sites or void spaces that should be used for CO_2_ transport. As a result, the transport due to solution-diffusion decreases [72,73]. Adding DEA also facilitates CO_2_ transportation through membranes. Facilitated CO_2_ transport is a globally non-reactive system, i.e. a system that acts strictly as a passive transport medium and not as a chemical reactor under stable conditions [74]. At a steady state, CO_2_ transport is mediated by the presence of DEA shuttling back and forth across membrane thickness. DEA on the membrane feed side responds with CO_2_ to species of carbamates and protonated amines. These species then spread to the membrane's permeate side, where the desorption or reverse reaction is high, releasing CO_2_. Also, DEA is produced and cycles back to the feed side, where the process is repeated [75].

The effect of DEA concentration on permeance has been well described in the cited literature [75]. It was found that at 20–30 wt% DEA concentration, the permeance of CO_2_ did not greatly increase. This is due to the trade-off between the favourable facilitation effect of high DEA concentration and the reduction of both ionic species diffusivity and CO_2_ solubility at high DEA concentrations. Also, the permeance is decreased with the increase of amine concentration because the ionic strength of the membranes increases at higher DEA concentration. Therefore, in this study, DEA concentration was limited to 15 wt%.

### 3.6. Comparison of results with literature

The CO_2_-CH_4_ separation factor of investigated composite amine mixed matrix was compared with the results reported in the literature, table 4. It is worth mentioning that no study is reported in the literature on composite amine MMM for CO_2_-CH_4_ separation. Hence, the comparison is made with composite MMMs. It shows that the addition of DEA as a carrier enhanced the performance of membrane and non-woven fabric as a support to improve the stability of membranes at high pressure. It was found that the performance of amine composite MMM is comparable with the literature.

**Table 4.** Performance comparison with literature data for CO_2_-CH_4_ separation.

polymer	molecular sieve	solvent/additive	pressure (bar)	CO_2_/CH_4_ selectivity	reference
PES	MWCNT	NMP	4	19.57	[76]
Elvaloy4170	functionalized multi-walled carbon nanotubes (3 wt%)	toluene	2	6.18	[77]
Pebax	—	glycerol triacetate (GTA) 40 wt%	4	approximately 13	[78]
PES	CMS (10 wt%)	NMP/15 wt% DEA	30	16.04	this study

## 4. Performance evaluation of amine composite mixed matrix membranes by Maxwell model

The Maxwell model is selected because of its mathematical simplicity and capability to integrate and account for all the physical components of MMMs (such as matrix and filler permeability, filler's volume fraction, where the last parameter plays a critical role in membrane performance). Moreover, it is easy to compute and uses a lesser number of assumptions than other similar models for MMMs [79–81]. However, the Maxwell model is unable to incorporate the effect of alkanolamine in its conventional form. Therefore, a slight modification was performed that allows the Maxwell model to include the effect of alkanolamine concentration in the performance of the alkanolamine based MMMs. In the Maxwell model, the volume fraction of filler (*φ_d_*) is a parameter that can play a vital role in predicting the performance of MMM. But in this study, the volume fraction of amine also played a key role in performance enhancement. Therefore, the volume fraction term needs to be modified to evaluate the performance of amine composite MMMs.

The experimental and literature parameters used for the model development are volume fraction of amine *φ_a_*, the volume fraction of dispersed phase (CMS) *φ_d_*, the combined volume fraction of filler and DEA $\varphi_{ad}^{**}$, facilitation parameter *F_c_,* estimated permeation of filler against pressure $P_{d}^{**}$, the permeability of polymer matrix *P_m_*_,_ and ratio of predicted filler permeability $P_{d}^{**}$ to the permeability of polymer matrix *P_m_*, $\lambda_{dm}^{**}$. The parameters listed above are related to each other as per the equations given below.

4.1 $$\varphi_{ad}^{**}=(\varphi_{a}+\varphi_{d})+F_{c}.$$

The volume fraction of CMS and amine is dependent on the experimental studies and has been determined from open literature for amine mixed matrix membranes. As for *F_c_*, the value was regressed to experimental values in this study by using the Delaunay Triangulation from the literature [27]. *F_c_* is required in this study to account for the shape factor of the amine in the membrane structure. Thus, $\varphi_{ad}^{**}$ is a combination of three parameters, as stated in equation (4.3).

Usually, the value $\lambda_{dm}^{**}$ is determined at a constant pressure. However, the operating pressure in this study varies. Therefore, the value is regressed to obtain the values at operating pressures used in this study. The fitting and optimization procedures were conducted on the Maxwell model against the high-pressure experimental data to produce the results for $\lambda_{dm}^{**}$. The *P_m_* value, on the other hand, is available through experimental work and varies with pressure. Once the $\lambda_{dm}^{**}$ values are determined, $P_{d}^{**}$ the predicted permeability of filler against pressure is obtained using equation (4.4).

4.2 $$\;\lambda_{dm}^{**}=\frac{\;P_{d}^{**}\;}{P_{m}}.$$

**Figure 10.**
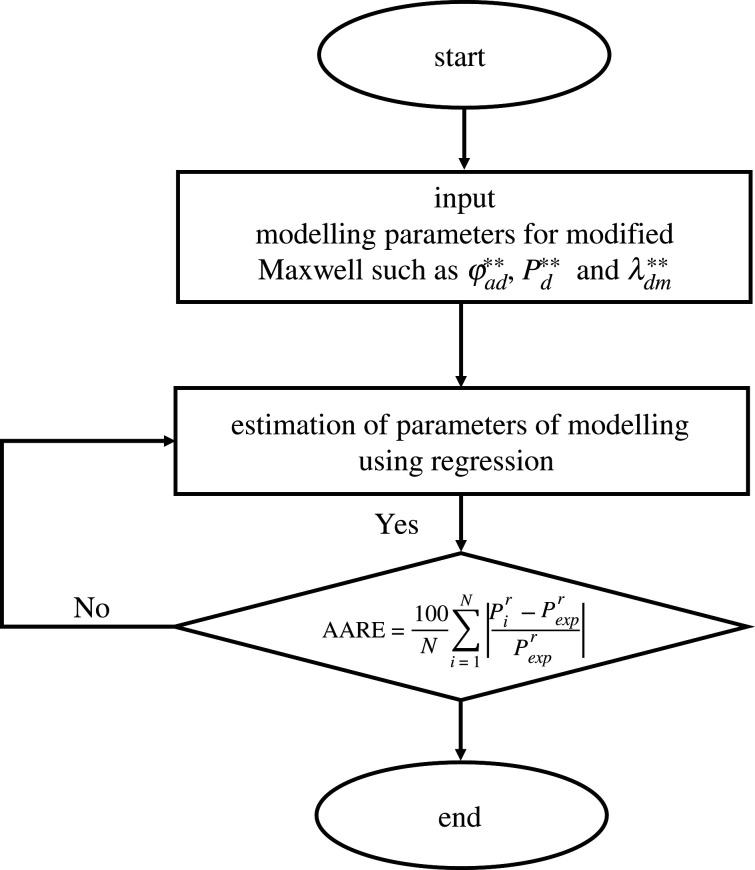
Flow chart for the modelling of amine composite MMMs at high pressure.

The addition of the new parameter $\varphi_{ad}^{**}$ in the basic Maxwell equation, based on the electrical or thermal conductivity model, leads to the modified Maxwell equation. Applying all the parameters in equation (4.3) results in determining the relative permeability through modelling. Figure 10 provides a graphical representation of the algorithm used for the modelling.

4.3 $$P_{r}=\frac{2(1-\varphi_{ad}^{**})+(1+2\varphi_{ad}^{**})\lambda_{dm}^{**}}{(2+\varphi_{ad}^{**})+(1-\varphi_{ad}^{**})\lambda_{dm}^{**}}.$$

The percent absolute average relative error (AARE%) between the *P_r_*_(cal)_ and experimental relative permeability *P_r_*_(exp)_ values were determined by equation (4.6).

4.4 $$\text{AARE\%}=\;\frac{100}{N}\overset{N}{\underset{i=1}{\sum }}\left|\frac{P_{i}^{r}-P_{\exp}^{r}}{P_{\exp}^{r}}\right|.$$

**Figure 11.**
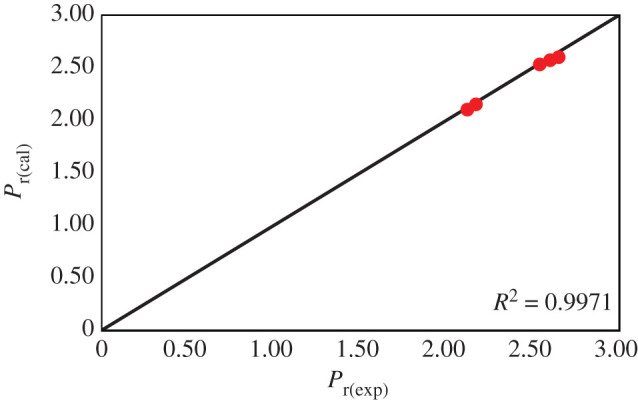
CO_2_ relative permeability (experimental) versus relative permeability (calculated) by the modified Maxwell model.

The model parameters of equation (4.3) are tabulated in table 5. The relative permeability was calculated by putting estimated model parameters for 10 wt% CMS loading and 15 wt% addition of DEA in polymer matrix for the pressure range of 10–30 bar in equation (4.3). It was noted that the calculated relative permeability is in good agreement with experimental relative permeabilities. The AARE% is in the range of 0.39 to 1.89%. Moreover, the experimental and calculated relative permeability was compared to graphically and presented in figure 11. It has shown a good agreement with *R*^2^ = 0.99.

**Table 5.** Estimated model parameters for fitting the high-pressure experimental data of CO_2_ separation through the amine composite mixed matrix membranes.

pressure (bar)	*φ_a_*	*φ_d_*	CO_2_ permeance (GPU)		*Fc* [27]	$\varphi_{ad}^{**}$	$\lambda_{dm}^{**}$	$P_{d}^{**}$	relative permeability		AARE %
			CM	CM-C10D15					*P_r_*_(exp)_	*P_r_*_(cal)_	
10	0.05	0.13	180.5	469.31	0.27	0.45	10.66	1924.45	2.60	2.57	1.15
15			161.83	411.67			10.07	1630.05	2.54	2.53	0.39
20			143.66	380.52			11.24	1615.08	2.65	2.60	1.89
25			137.35	297.97			5.83	800.50	2.17	2.15	0.92
30			142.21	300.84			5.44	773.90	2.12	2.10	0.94

A very small difference was found in the values of calculated and experimental values of relative permeabilities. This difference is because the basic Maxwell model was developed for the prediction of electrical conductivity and later further modified for the mixed matrix membranes. The Maxwell model is developed for two (polymer and filler) phase system and low-pressure applications. In this study, the developed amine composite mixed matrix membranes have three (polymer + filler + amine) phases for high-pressure gas transport. Another reason could be the gas transport mechanism [23,82,83]. The developed composite amine mixed matrix membranes follow the solution diffusion through the polymer matrix, molecular sieving through CMS, and facilitated transport through DEA [84].

On the other hand, the Maxwell model obeys the solution diffusion and molecular sieving mechanisms. Beyond these reasons, the modified Maxwell model gives a good prediction of gas transport with small AARE%. In figure 11, the *R*^2^ value shows that the modified model can predict the performance of composite amine mixed matrix membranes satisfactory for carbon dioxide.

So, based on results, it is concluded that the modified Maxwell model is working satisfactorily at high pressure 10 wt% of CMS with constant concertation of DEA. But more studies are needed to understand the detailed geometry of composite amine mixed matrix membranes.

## 5. Conclusion

Asymmetric amine composite mixed matrix membranes with CMS, DEA and PES were synthesized and tested for CO_2_-CH_4_ separation. The addition of DEA has enhanced CO_2_-CH_4_ separation performance, and a more than twofold increment has been observed in CO_2_ permeance. Similarly, approximately threefold increment has been found in CO_2_-CH_4_ selectivity as compared to the pure PES membrane. The FESEM analysis confirmed the smooth and defect-free surface and typical finger-like and sponge-like pore structures, which help to reduce the transport resistance and to provide enough mechanical strength, respectively. The robust mechanical properties of the amine composite MMMs may be attributed to the strong interactions between the PES matrix, CMS, and DEA, in addition to homogeneous dispersion and adhesion of CMS. The modified Maxwell model, after including the optimized $\lambda_{dm}^{**}$ along with facilitation factor *F_c_* and predicted permeability of dispersed phase $P_{d}^{**}$, has been developed to predict the performance of composite amine MMMs at high pressure. The results exhibited that the model predicted the relative permeability with *R*^2^ = 0.99.

## Supplementary Material

Figure S1

Reviewer comments

## Data accessibility

All data associated with the study, including raw gas separation data, have been deposited in the Dryad Digital Repository https://doi.org/10.5061/dryad.dfn2z34xv. Additionally, the FTIR spectra for table 2 has been given as electronic supplementary material with the manuscript.

## References

[1] Robeson LM 1991 Correlation of separation factor versus permeability for polymeric membranes. J. Membr. Sci. 62, 165–185. (10.1016/0376-7388(91)80060-J)

[2] Scott K 1995 Handbook of industrial membranes. Oxford, UK: Elsevier.

[3] Strathmann HJAj 2001 Membrane separation processes: current relevance and future opportunities. AIChE Journal 47, 1077–1087. (10.1002/aic.690470514)

[4] Madzarevic ZP, Shahid S, Nijmeijer K, Dingemans TJ 2019 The role of ortho-, meta- and para-substitutions in the main-chain structure of poly(etherimide)s and the effects on CO_2_/CH_4_ gas separation performance. Sep. Purif. Technol. 210, 242–250. (10.1016/j.seppur.2018.08.006)

[5] Ishaq S, Tamime R, Bilad MR, Khan AL 2019 Mixed matrix membranes comprising of polysulfone and microporous Bio-MOF-1: preparation and gas separation properties. Sep. Purif. Technol. 210, 442–451. (10.1016/j.seppur.2018.08.031)

[6] Jiang Y, Liu C, Caro J, Huang A 2019 A new UiO-66-NH2 based mixed-matrix membranes with high CO_2_/CH_4_ separation performance. Microporous Mesoporous Mater. 274, 203–211. (10.1016/j.micromeso.2018.08.003)

[7] Cheng Y et al. 2019 Mixed matrix membranes containing MOF@COF hybrid fillers for efficient CO_2_/CH_4_ separation. J. Membr. Sci. 573, 97–106. (10.1016/j.memsci.2018.11.060)

[8] Anastasiou S, Bhoria N, Pokhrel J, Kumar Reddy KS, Srinivasakannan C, Wang K, Karanikolos GN 2018 Metal-organic framework/graphene oxide composite fillers in mixed-matrix membranes for CO_2_ separation. Mater. Chem. Phys. 212, 513–522. (10.1016/j.matchemphys.2018.03.064)

[9] Li H, Tuo L, Yang K, Jeong H-K, Dai Y, He G, Zhao W 2016 Simultaneous enhancement of mechanical properties and CO_2_ selectivity of ZIF-8 mixed matrix membranes: interfacial toughening effect of ionic liquid. J. Membr. Sci. 511, 130–142. (10.1016/j.memsci.2016.03.050)

[10] Cheng Y, Zhai L, Ying Y, Wang Y, Liu G, Dong J, Ng DZL, Khan SA, Zhao D 2019 Highly efficient CO_2_ capture by mixed matrix membranes containing three-dimensional covalent organic framework fillers. J. Mater. Chem. A 7, 4549–4560. (10.1039/C8TA10333J)

[11] Mohammadnezhad F, Feyzi M, Zinadini S 2019 A novel Ce-MOF/PES mixed matrix membrane; synthesis, characterization and antifouling evaluation. J. Ind. Eng. Chem. 71, 99–111. (10.1016/j.jiec.2018.09.032)

[12] Azari M, Sadeghi M, Aroon M, Matsuura T 2019 Polyurethane mixed matrix membranes for gas separation: a systematic study on effect of SiO_2_/TiO_2_ nanoparticles. J. Membr. Sci. Res. 5, 33–43.

[13] Li P, Chen HZ, Chung T-S 2013 The effects of substrate characteristics and pre-wetting agents on PAN–PDMS composite hollow fiber membranes for CO_2_/N_2_ and O_2_/N_2_ separation. J. Membr. Sci. 434, 18–25. (10.1016/j.memsci.2013.01.042)

[14] Liang CZ, Yong WF, Chung T-S 2017 High-performance composite hollow fiber membrane for flue gas and air separations. J. Membr. Sci. 541, 367–377. (10.1016/j.memsci.2017.07.014)

[15] Liang CZ, Liu JT, Lai J-Y, Chung T-S 2018 High-performance multiple-layer PIM composite hollow fiber membranes for gas separation. J. Membr. Sci. 563, 93–106. (10.1016/j.memsci.2018.05.045)

[16] Norahim N, Faungnawakij K, Quitain AT, Klaysom C 2019 Composite membranes of graphene oxide for CO_2_/CH_4_ separation. J. Chem. Technol. Biotechnol. 94, 2783–2791. (10.1002/jctb.5999)

[17] Khalilinejad I, Sanaeepur H, Kargari A 2015 Preparation of poly(ether-6-block amide)/PVC thin film composite membrane for CO_2_ Separation: effect of top layer thickness and operating parameters. J. Membr. Sci. Res. 1, 124–129.

[18] Mozafari M, Abedini R, Rahimpour A 2018 Zr-MOFs-incorporated thin film nanocomposite Pebax 1657 membranes dip-coated on polymethylpentyne layer for efficient separation of CO_2_/CH_4_. J. Mater. Chem. A 6, 12 380–12 392. (10.1039/C8TA04806A)

[19] Maghami S, Sadeghi M, Mehrabani-Zeinabad A 2017 Recognition of polymer-particle interfacial morphology in mixed matrix membranes through ideal permeation predictive models. Polym. Test. 63, 25–37. (10.1016/j.polymertesting.2017.07.021)

[20] Gheimasi KM, Mohammadi T, Bakhtiari O 2015 Using a new model for prediction of gas permeability through MMMs: considering effects of particles shape, polymer chain rigidification, partial pore blockage, and void formation. Sep. Sci. Technol. 50, 2384–2395.

[21] Maghami S, Mehrabani-Zeinabad A, Sadeghi M, Sánchez-Laínez J, Zornoza B, Téllez C, Coronas J 2019 Mathematical modeling of temperature and pressure effects on permeability, diffusivity and solubility in polymeric and mixed matrix membranes. Chem. Eng. Sci. 205, 58–73. (10.1016/j.ces.2019.04.037)

[22] Mohshim DF, Mukhtar H, Dutta BK, Man Z 2019 Predicting CO_2_ permeation through an enhanced ionic liquid mixed matrix membrane (IL3M). Int. J. Chem. Eng. 2019, 1–10. (10.1155/2019/9525783)

[23] Vinh-Thang H, Kaliaguine S 2013 Predictive models for mixed-matrix membrane performance: a review. Chem. Rev. 113, 4980–5028. (10.1021/cr3003888) 23548158

[24] Bakonyi P, Nemestóthy N, Bélafi-Bakó K 2013 Biohydrogen purification by membranes: an overview on the operational conditions affecting the performance of non-porous, polymeric and ionic liquid based gas separation membranes. Int. J. Hydrogen Energy 38, 9673–9687. (10.1016/j.ijhydene.2013.05.158)

[25] Nasir R, Mukhtar H, Man Z, Dutta BK, Shaharun MS, Abu Bakar MZ 2015 Mixed matrix membrane performance enhancement using alkanolamine solution. J. Membr. Sci. 483, 84–93. (10.1016/j.memsci.2015.02.041)

[26] Maxwell J 1954 *A treatise on electricity and magnetism*, vol. 1. New York, NY: Dover Publications Inc.

[27] Nasir R, Mukhtar H, Man Z 2016 Prediction of gas transport across amine mixed matrix membranes with ideal morphologies based on the Maxwell model. RSC Adv. 6, 30 130–30 138. (10.1039/C5RA27756F)

[28] Belgacema IB, Bouhamedb H, Loulerguec P, Szymczykc A, Khemakhema S 2019 Elaboration and characterization of novel PES/nanocomposites mixed matrix membranes. Desalination Water Treat. 169, 102–113. (10.5004/dwt.2019.24763)

[29] Haul R 1993 J. Kärger, D. M. Ruthven: Diffusion in Zeolites and other Microporous Solids, J. Wiley & Sons INC, New York 1992. ISBN 0-471-50907-8. 605 Seiten, Preis: £ 117. Berichte der Bunsengesellschaft für physikalische Chemie 97, 146–147. (10.1002/bbpc.19930970130)

[30] Nasir R, Mukhtar H, Shima Shaharun M, Man Z 2015 Effect of carbon molecular sieve (CMS) concentration on mixed matrix membranes (MMMs) performance for carbon dioxide removal. Appl. Mech. Mater. 754, 869–873. (10.4028/www.scientific.net/AMM.754-755.869)

[31] Nasir R. 2016 *Synthesis, characterization and performance evaluation of amine mixed matrix membranes for CO_2_/CH_4_ separation*. Doctoral dissertation. Malaysia: Universiti Teknologi PETRONAS.

[32] Pinnau I, Wind J, Peinemann KV 1990 Ultrathin multicomponent poly(ether sulfone) membranes for gas separation made by dry/wet phase inversion. Ind. Eng. Chem. Res. 29, 2028–2032. (10.1021/ie00106a009)

[33] Lin D-J, Chang C-L, Lee C-K, Cheng L-P 2006 Preparation and characterization of microporous PVDF/PMMA composite membranes by phase inversion in water/DMSO solutions. Eur. Polym. J. 42, 2407–2418. (10.1016/j.eurpolymj.2006.05.008)

[34] Abdul Mannan H, Yih TM, Nasir R, Muhktar H, Mohshim DF 2019 Fabrication and characterization of polyetherimide/polyvinyl acetate polymer blend membranes for CO_2_/CH_4_ separation. Polym. Eng. Sci. 59, E293–E301. (10.1002/pen.24945)

[35] Van de Witte P, Dijkstra P, Van den Berg J, Feijen J 1996 Phase separation processes in polymer solutions in relation to membrane formation. J. Membr. Sci. 117, 1–31. (10.1016/0376-7388(96)00088-9)

[36] Dong X, Al-Jumaily A, Escobar IC 2018 Investigation of the use of a bio-derived solvent for non-solvent-induced phase separation (NIPS) fabrication of polysulfone membranes. Membranes 8, 23 (10.3390/membranes8020023) PMC6026890 29735925

[37] Frommer MA, Lancet D 1972 Reverse osmosis membrane research. Berlin, Germany: Springer, pp. 85–110.

[38] Eke J, Mills PA, Page JR, Wright GP, Tsyusko OV, Escobar IC 2020 Nanohybrid membrane synthesis with phosphorene nanoparticles: a study of the addition, stability and toxicity. Polymers 12, 1555 (10.3390/polym12071555) PMC7408299 32674304

[39] Kim I-C, Yun H-G, Lee K-H 2002 Preparation of asymmetric polyacrylonitrile membrane with small pore size by phase inversion and post-treatment process. J. Membr. Sci. 199, 75–84. (10.1016/S0376-7388(01)00680-9)

[40] Salim NE et al. 2018 Performance of PES/LSMM-OGCN photocatalytic membrane for phenol removal: effect of OGCN loading. Membranes 8, 42 (10.3390/membranes8030042) PMC6161286 29997383

[41] Eykens L, De Sitter K, Stoops L, Dotremont C, Pinoy L, Van der Bruggen B 2017 Development of polyethersulfone phase-inversion membranes for membrane distillation using oleophobic coatings. J. Appl. Polym. Sci. 134, 45516 (10.1002/app.45516)

[42] Zinadini S, Zinatizadeh AA, Rahimi M, Vatanpour V, Zangeneh H 2014 Preparation of a novel antifouling mixed matrix PES membrane by embedding graphene oxide nanoplates. J. Membr. Sci. 453, 292–301. (10.1016/j.memsci.2013.10.070)

[43] Vankelecom IFJ, Scheppers E, Heus R, Uytterhoeven JB 1994 Parameters influencing zeolite incorporation in PDMS membranes. J. Phys. Chem. 98, 12 390–12 396. (10.1021/j100098a038)

[44] Nasrollahi N, Vatanpour V, Aber S, Mahmoodi NM 2018 Preparation and characterization of a novel polyethersulfone (PES) ultrafiltration membrane modified with a CuO/ZnO nanocomposite to improve permeability and antifouling properties. Sep. Purif. Technol. 192, 369–382. (10.1016/j.seppur.2017.10.034)

[45] Bahri SS, Harun Z, Salleh WNW, Nur M, Abd Kadir H, Sazali N, Hussin R, Basri H 2019 The influence of Fe doped TiO_2_ as inorganic additive on the properties of polysulfone ultrafitration membrane. Malays. J. Fundam. Appl. Sci. 15, 725–730.

[46] Yong HH, Park HC, Kang YS, Won J, Kim WN 2001 Zeolite-filled polyimide membrane containing 2,4,6-triaminopyrimidine. J. Membr. Sci. 188, 151–163. (10.1016/S0376-7388(00)00659-1)

[47] Ma Y, Shi F, Ma J, Wu M, Zhang J, Gao C 2011 Effect of PEG additive on the morphology and performance of polysulfone ultrafiltration membranes. Desalination 272, 51–58. (10.1016/j.desal.2010.12.054)

[48] Vilakati GD, Hoek EM, Mamba BB 2014 Probing the mechanical and thermal properties of polysulfone membranes modified with synthetic and natural polymer additives. Polym. Test. 34, 202–210. (10.1016/j.polymertesting.2014.01.014)

[49] Pavia DL, Lampman GM, Kriz GS, Vyvyan JA 2008 Introduction to spectroscopy. Belmont, CA: Cengage Learning.

[50] Adewole JK, Ahmad AL, Ismail S, Leo CP, Sultan AS 2015 Comparative studies on the effects of casting solvent on physico-chemical and gas transport properties of dense polysulfone membrane used for CO_2_/CH_4_ separation. J. Appl. Polym. Sci. 132, 42205. (10.1002/app.42205)

[51] Ghaemi N, Madaeni SS, Daraei P, Rajabi H, Shojaeimehr T, Rahimpour F, Shirvani B 2015 PES mixed matrix nanofiltration membrane embedded with polymer wrapped MWCNT: fabrication and performance optimization in dye removal by RSM. J. Hazard. Mater. 298, 111–121. (10.1016/j.jhazmat.2015.05.018) 26022851

[52] Vu DQ, Koros WJ, Miller SJ 2003 Mixed matrix membranes using carbon molecular sieves. J. Membr. Sci. 211, 311–334. (10.1016/S0376-7388(02)00429-5)

[53] Eisenberg A, Hird B, Moore R 1990 A new multiplet-cluster model for the morphology of random ionomers. Macromolecules 23, 4098–4107. (10.1021/ma00220a012)

[54] Pradhan DK, Choudhary R, Samantaray B, Karan N, Katiyar R 2007 Effect of plasticizer on structural and electrical properties of polymer nanocompsoite electrolytes. Int. J. Electrochem. Sci. 2, 861–871.

[55] Şen D, Kalıpçılar H, Yilmaz L 2007 Development of polycarbonate based zeolite 4A filled mixed matrix gas separation membranes. J. Membr. Sci. 303, 194–203. (10.1016/j.memsci.2007.07.010)

[56] Manawi YM, Wang K, Kochkodan V, Johnson DJ, Atieh MA, Khraisheh MK 2018 Engineering the surface and mechanical properties of water desalination membranes using ultralong carbon nanotubes. Membranes 8, 106 (10.3390/membranes8040106) PMC6315779 30428620

[57] Lord JD, Morrell R 2010 Elastic modulus measurement—obtaining reliable data from the tensile test. Metrologia 47, S41–S49. (10.1088/0026-1394/47/2/S05)

[58] Flyagina IS, Mahdi E, Titov K, Tan J-C 2017 Thermo-mechanical properties of mixed-matrix membranes encompassing zeolitic imidazolate framework-90 and polyvinylidine difluoride: ZIF-90/PVDF nanocomposites. APL Mater. 5, 086104 (10.1063/1.4986565)

[59] Mohanapriya S, Bhat S, Sahu AK, Pitchumani S, Sridhar P, Shukla AK 2009 A new mixed-matrix membrane for DMFCs. Energy Environ. Sci. 2, 1210–1216. (10.1039/b909451b)

[60] Rekik SB, Gassara S, Bouaziz J, Deratani A, Baklouti S 2019 Enhancing hydrophilicity and permeation flux of chitosan/kaolin composite membranes by using polyethylene glycol as porogen. Appl. Clay Sci. 168, 312–323. (10.1016/j.clay.2018.11.029)

[61] Zambare RS, Dhopte KB, Patwardhan AV, Nemade PR 2017 Polyamine functionalized graphene oxide polysulfone mixed matrix membranes with improved hydrophilicity and anti-fouling properties. Desalination 403, 24–35. (10.1016/j.desal.2016.02.003)

[62] Ali M, Zafar M, Jamil T, Butt MTZ 2011 Influence of glycol additives on the structure and performance of cellulose acetate/zinc oxide blend membranes. Desalination 270, 98–104. (10.1016/j.desal.2010.11.027)

[63] Saedi S, Madaeni SS, Seidi F, Shamsabadi AA, Laki S 2014 Fixed facilitated transport of CO_2_ through integrally-skinned asymmetric polyethersulfone membrane using a novel synthesized Poly (acrylonitrile-co-N, *N*-dimethylaminopropyl acrylamide). Chem. Eng. J. 236, 263–273. (10.1016/j.cej.2013.09.092)

[64] Visser T, Koops GH, Wessling M 2005 On the subtle balance between competitive sorption and plasticization effects in asymmetric hollow fiber gas separation membranes. J. Membr. Sci. 252, 265–277. (10.1016/j.memsci.2004.12.015)

[65] Khan AL, Li X, Vankelecom IF 2011 SPEEK/Matrimid blend membranes for CO_2_ separation. J. Membr. Sci. 380, 55–62. (10.1016/j.memsci.2011.06.030)

[66] Ismail A, Yaacob N 2006 Performance of treated and untreated asymmetric polysulfone hollow fiber membrane in series and cascade module configurations for CO_2_/CH_4_ gas separation system. J. Membr. Sci. 275, 151–165. (10.1016/j.memsci.2005.09.014)

[67] Saberi M, Dadkhah A, Hashemifard S 2016 Modeling of simultaneous competitive mixed gas permeation and CO_2_ induced plasticization in glassy polymers. J. Membr. Sci. 499, 164–171. (10.1016/j.memsci.2015.09.044)

[68] Stannett V 1978 The transport of gases in synthetic polymeric membranes — an historic perspective. J. Membr. Sci. 3, 97–115. (10.1016/S0376-7388(00)83016-1)

[69] Ahmad MZ, Peters TA, Konnertz NM, Visser T, Téllez C, Coronas J, Fila V, de Vos WM, Benes NE 2020 High-pressure CO_2_/CH4 separation of Zr-MOFs based mixed matrix membranes. Sep. Purif. Technol. 230, 115858 (10.1016/j.seppur.2019.115858)

[70] Wiryoatmojo AS, Mukhtar H, Man Z 2009 Development of polysulfone-carbon molecular sieves mixed matrix membranes for CO_2_ removal from natural gas. In *Chemical, Biological and Environmental Engineering: Proceedings of the International Conference on Cbee 2009*, *Singapore, 9–11 October 2009*, p. 249. Singapore; Hackensack, NJ: World Scientific Publishing.

[71] Rafizah W, Ismail A 2008 Effect of carbon molecular sieve sizing with poly(vinyl pyrrolidone) K-15 on carbon molecular sieve–polysulfone mixed matrix membrane. J. Membr. Sci. 307, 53–61. (10.1016/j.memsci.2007.09.007)

[72] Francisco GJ, Chakma A, Feng X 2010 Separation of carbon dioxide from nitrogen using diethanolamine-impregnated poly(vinyl alcohol) membranes. Sep. Purif. Technol. 71, 205–213. (10.1016/j.seppur.2009.11.023)

[73] Francisco GJ, Chakma A, Feng X 2007 Membranes comprising of alkanolamines incorporated into poly(vinyl alcohol) matrix for CO2/N2 separation. J. Membr. Sci. 303, 54–63. (10.1016/j.memsci.2007.06.065)

[74] Schultz JS, Goddard JD, Suchdeo SR 1974 Facilitated transport via carrier-mediated diffusion in membranes: Part I. Mechanistic aspects, experimental systems and characteristic regimes. AlChE J. 20, 417–445. (10.1002/aic.690200302)

[75] Francisco GJ 2006 Separation of carbon dioxide from nitrogen using poly (vinyl alcohol)-amine blend membranes. Doctoral dissertation. Waterloo, Canada: University of Waterloo.

[76] Ismail A, Rahim N, Mustafa A, Matsuura T, Ng B, Abdullah S, Hashemifard S 2011 Gas separation performance of polyethersulfone/multi-walled carbon nanotubes mixed matrix membranes. Sep. Purif. Technol. 80, 20–31. (10.1016/j.seppur.2011.03.031)

[77] Ranjbaran F, Omidkhah MR, Ebadi Amooghin A 2015 The novel Elvaloy4170/functionalized multi-walled carbon nanotubes mixed matrix membranes: fabrication, characterization and gas separation study. J. Taiwan Inst. Chem. Eng. 49, 220–228. (10.1016/j.jtice.2014.11.032)

[78] Rabiee H, Soltanieh M, Mousavi SA, Ghadimi A 2014 Improvement in CO_2_/H_2_ separation by fabrication of poly(ether-b-amide6)/glycerol triacetate gel membranes. J. Membr. Sci. 469, 43–58. (10.1016/j.memsci.2014.06.026)

[79] Shimekit B, Mukhtar H, Murugesan T 2011 Prediction of the relative permeability of gases in mixed matrix membranes. J. Membr. Sci. 373, 152–159. (10.1016/j.memsci.2011.02.038)

[80] Pal R 2008 Permeation models for mixed matrix membranes. J. Colloid Interface Sci. 317, 191–198. (10.1016/j.jcis.2007.09.032) 17935727

[81] Rybak A, Rybak A, Sysel P 2018 Modeling of gas permeation through mixed-matrix membranes using novel computer application MOT. Appl. Sci. 8, 1166 (10.3390/app8071166)

[82] Gonzo EE, Parentis ML, Gottifredi JC 2006 Estimating models for predicting effective permeability of mixed matrix membranes. J. Membr. Sci. 277, 46–54. (10.1016/j.memsci.2005.10.007)

[83] Petropoulos J 1985 A comparative study of approaches applied to the permeability of binary composite polymeric materials. J. Polym. Sci. Polym. Phys. Ed. 23, 1309–1324. (10.1002/pol.1985.180230703)

[84] Nasir R, Mukhtar H, Man Z, Shaharun MS, Abu Bakar MZ 2015 Effect of fixed carbon molecular sieve (CMS) loading and various di-ethanolamine (DEA) concentrations on the performance of a mixed matrix membrane for CO_2_/CH_4_ separation. RSC Adv. 5, 60 814–60 822. (10.1039/C5RA09015F)

