# Supplementary figures and images for: Composite amine mixed matrix membranes for high-pressure CO2-CH4 separation: synthesis, characterization and performance evaluation
Source: R Soc Open Sci. 2020 Sep 9;7(9):200795. doi: 10.1098/rsos.200795 (PMC7540797; doi:10.1098/rsos.200795)

# Membrane ID = CM

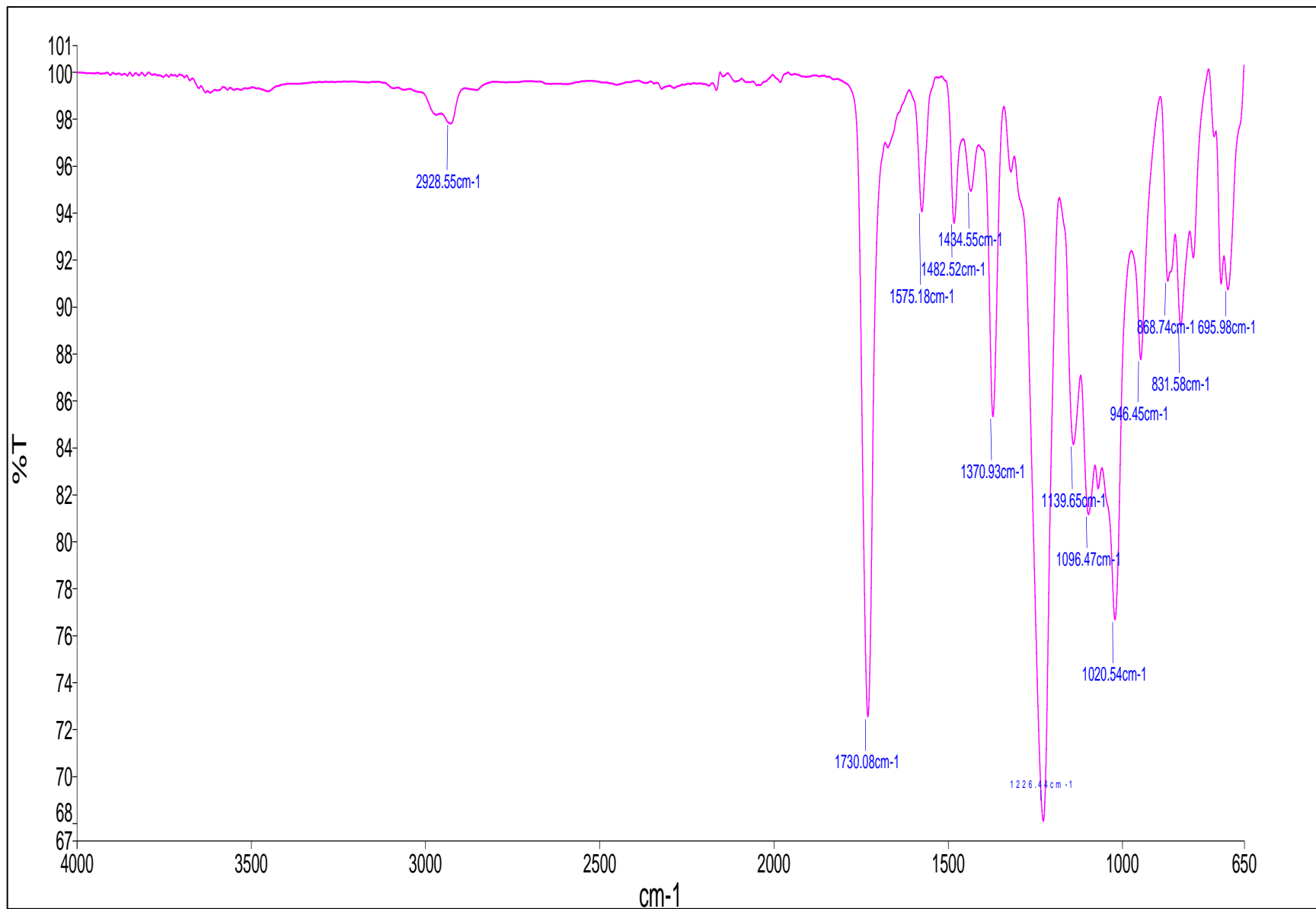

# Membrane ID = CM-C10

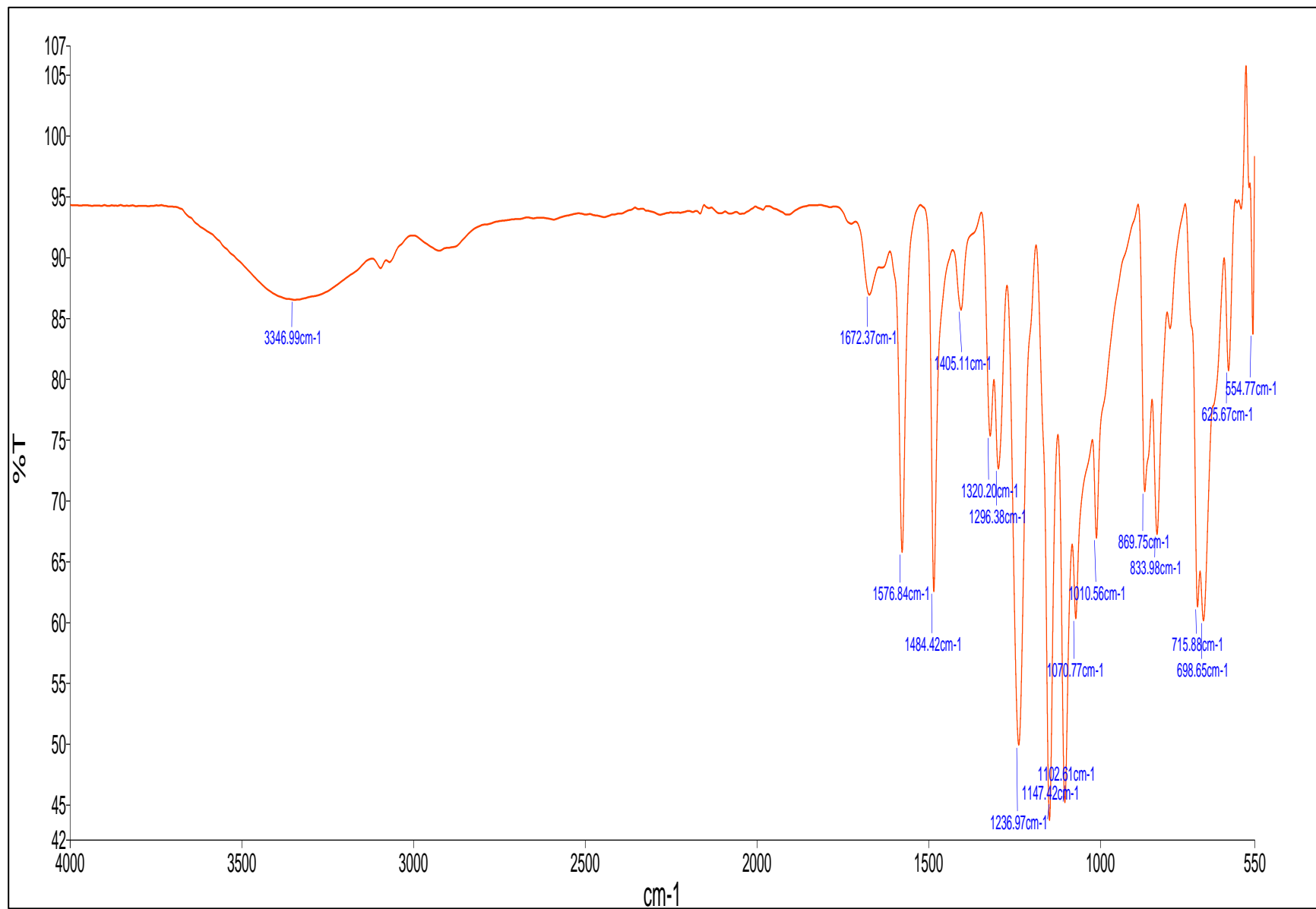

# Membrane ID = CM-C10D5

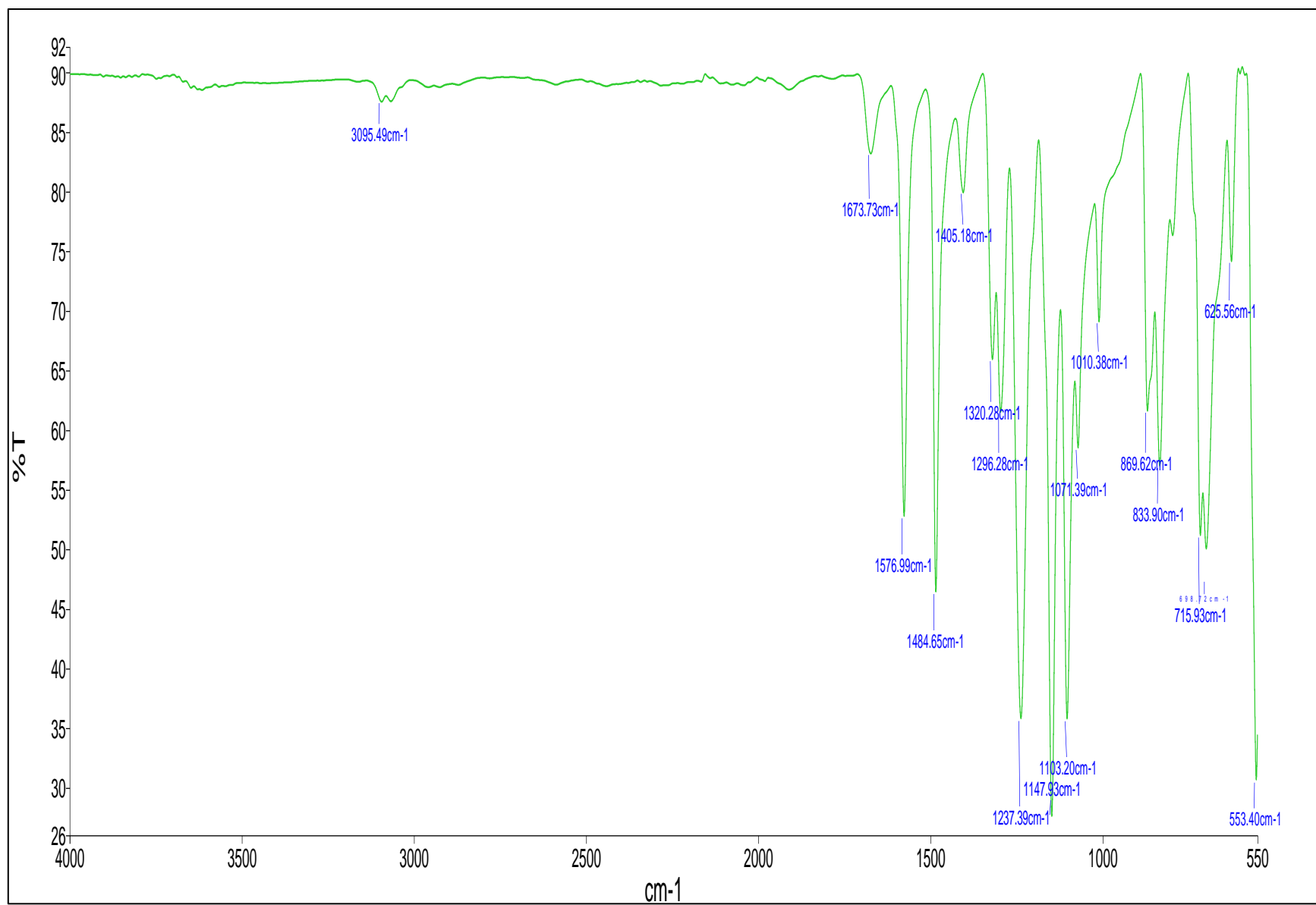

# Membrane ID = CM-C10D10

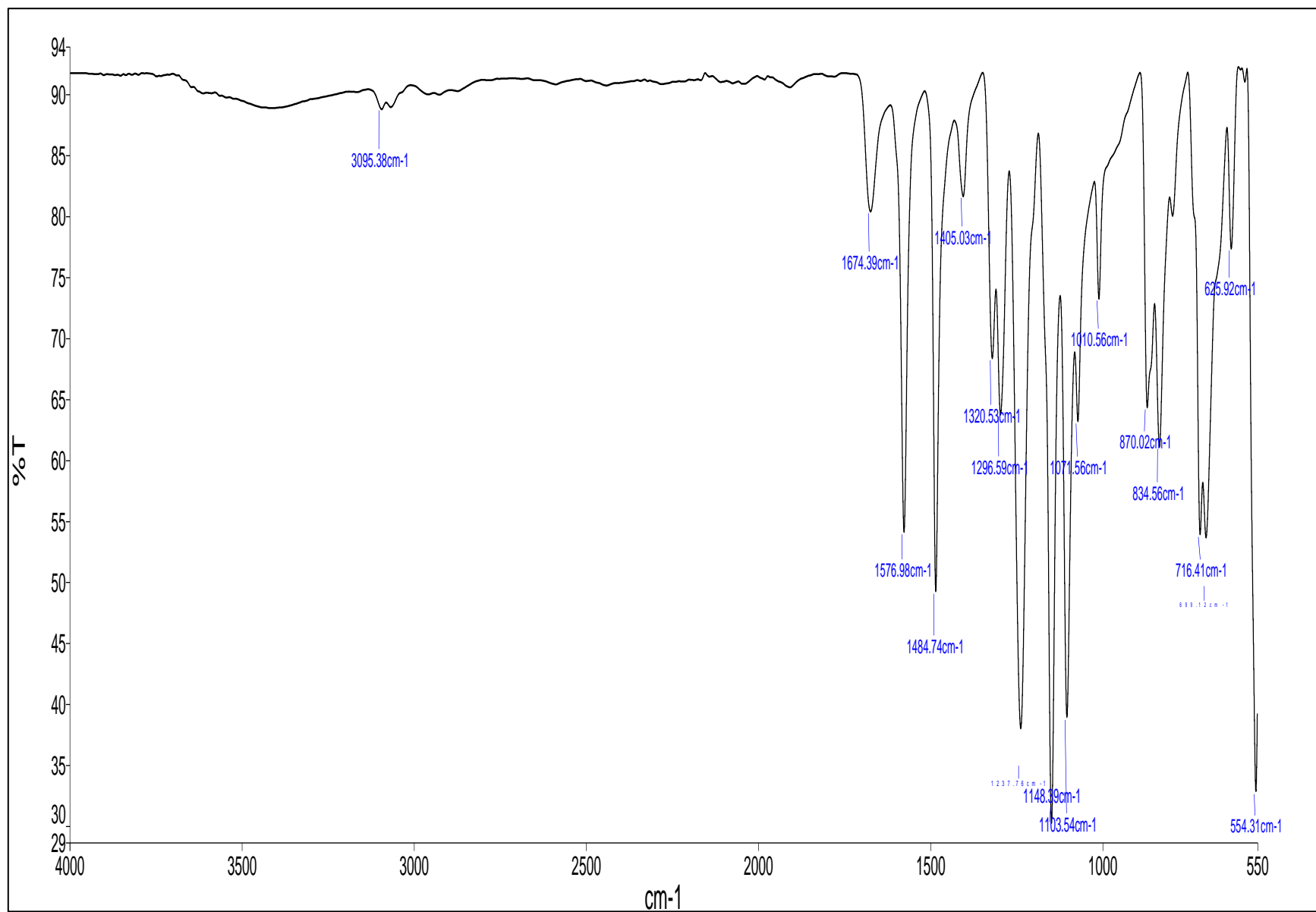

# Membrane ID = CM-C10D15

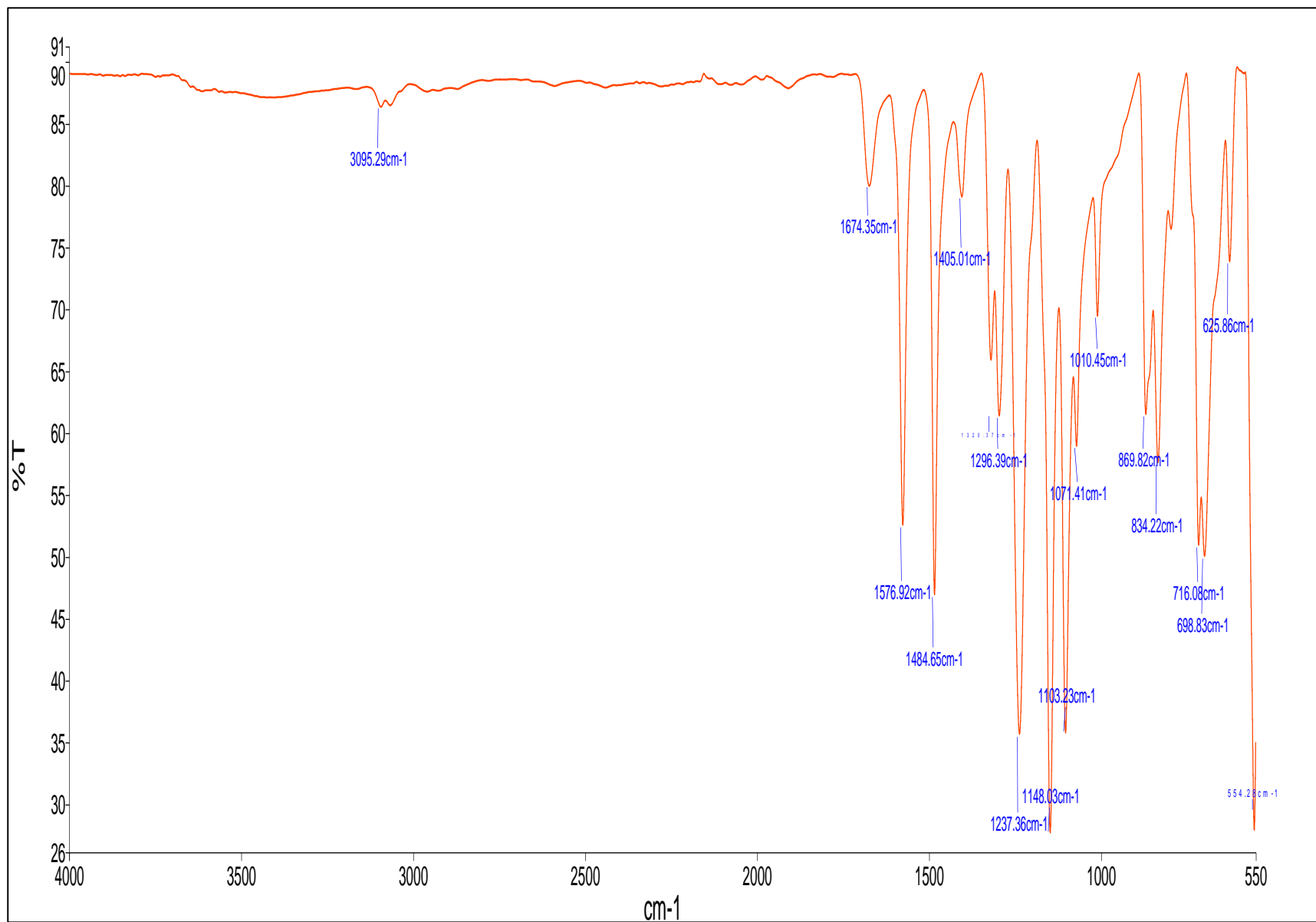

Supplement: Figure S1 [file rsos200795supp1.pdf]
